# PPAR-*γ* Agonist Pioglitazone Restored Mouse Liver mRNA Expression of Clock Genes and Inflammation-Related Genes Disrupted by Reversed Feeding

**DOI:** 10.1155/2022/7537210

**Published:** 2022-05-26

**Authors:** T. Fedchenko, O. Izmailova, V. Shynkevych, O. Shlykova, I. Kaidashev

**Affiliations:** Poltava State Medical University, Ukraine

## Abstract

**Introduction:**

The master clock, which is located in the suprachiasmatic nucleus (SCN), harmonizes clock genes present in the liver to synchronize life rhythms and bioactivity with the surrounding environment. The reversed feeding disrupts the expression of clock genes in the liver. Recently, a novel role of PPAR-*γ* as a regulator in correlating circadian rhythm and metabolism was demonstrated. This study examined the influence of PPAR-*γ* agonist pioglitazone (PG) on the mRNA expression profile of principle clock genes and inflammation-related genes in the mouse liver disrupted by reverse feeding.

**Methods:**

Mice were randomly assigned to daytime-feeding and nighttime-feeding groups. Mice in daytime-feeding groups received food from 7 AM to 7 PM, and mice in nighttime-feeding groups received food from 7 PM to 7 AM. PG was administered in the dose of 20 mg/kg per os as aqueous suspension 40 *μ*l at 7 AM or 7 PM. Each group consisted of 12 animals. On day 8 of the feeding intervention, mice were sacrificed by cervical dislocation at noon (05 hours after light onset (HALO)) and midnight (HALO 17). Liver expressions of *Bmal1*, *Clock*, *Rev-erb alpha*, *Cry1*, *Cry2*, *Per1*, *Per2*, *Cxcl5*, *Nrf2*, and *Ppar-γ* were determined by quantitative reverse transcription PCR. Liver expression of PPAR-*γ*, pNF-*κ*B, and IL-6 was determined by Western blotting. Glucose, ceruloplasmin, total cholesterol, triglyceride concentrations, and ALT and AST activities were measured in sera by photometric methods. The null hypothesis tested was that PG and the time of its administration have no influence on the clock gene expression impaired by reverse feeding.

**Results:**

Administration of PG at 7 AM to nighttime-feeding mice did not reveal any influence on the expression of the clock or inflammation-related genes either at midnight or at noon. In the daytime-feeding group, PG intake at 7 PM led to an increase in *Per2* and *Rev-erb alpha* mRNA at noon, an increase in *Ppar-γ* mRNA at midnight, and a decrease in *Nfκb* (*p65*) mRNA at noon. In general, PG administration at 7 PM slightly normalized the impaired expression of clock genes and increased anti-inflammatory potency impaired by reversed feeding. This pattern was supported by biochemical substrate levels—glucose, total cholesterol, ALT, and AST activities. The decrease in NF-*κ*B led to the inhibition of serum ceruloplasmin levels as well as IL-6 in liver tissue. According to our data, PG intake at 7 PM exerts strong normalization of clock gene expression with a further increase in *Nrf2* and, especially, *Ppar-γ* and PPAR-*γ* expression with inhibition of *Nfκb* and pNF-*κ*B expression in daytime-feeding mice. These expression changes resulted in decreased hyperglycemia, hypercholesterolemia, ALT, and AST activities. Thus, PG had a potent chronopharmacological effect when administered at 7 PM to daytime-feeding mice.

**Conclusions:**

Our study indicates that reversed feeding induced the disruption of mouse liver circadian expression pattern of clock genes accompanied by increasing *Nfκb* and pNF-*κ*B and IL-6 expression and decreasing *Nrf2* and PPAR-*γ*. Administration of PG restored the clock gene expression profile and decreased *Nfκb*, pNF-*κ*B, and IL-6, as well as increased *Nrf2*, *Ppar-γ*, and PPAR-*γ* expression. PG intake at 7 PM was more effective than at 7 AM in reversed feeding mice.

## 1. Introduction

The master clock, which is located in the suprachiasmatic nucleus (SCN), harmonizes clock genes present in peripheral organs (e.g., the liver, kidneys, and heart) and controls the daily waking state, behavior, and physiological functions to synchronize life rhythms and bioactivity with the surrounding environment [1].

Master clock genes are mainly entrained by the light-dark cycle [1], whereas peripheral clock genes are entrained by feeding rhythms [2]. Shifting the light-dark cycle alters the rhythms of master clock genes, and changing the feeding rhythm alters the rhythms of peripheral clock genes. Such alterations result in the uncoupling of master and peripheral clock genes, which can lead to high blood pressure, sleep disorders, glucose intolerance [3], and exacerbated inflammatory response. Alteration of food availability demonstrates the hierarchy of the cell-intrinsic hepatocyte clock mechanism and the feeding environment [4]. The liver clock had a central role in nutrient processing and its prominent response to daytime feeding [5, 6]. Reversed feeding may lead to the exacerbation of inflammatory responses via downregulation of survival factors such as peroxisome proliferator-activated receptor *γ* coactivator 1*α* (PGC-1*α*) and SIRT1 [7], interacting with clock genes [8, 9]. It was shown that several inflammation-related genes are strongly associated with the molecular clock, such as the nuclear erythroid 2-related factor 2 (NRF2) [10, 11], transcriptional factor NF-*κ*B [12], and neutrophil directing chemokine (C-X-C motif) ligand 5 (Cxcl5) [13], as well as peroxisome proliferator receptor-*γ* (PPAR-*γ*) [14–16].

Agonist PPAR-*γ* pioglitazone (PG) has been shown to transform blood pressure from a nondipper to a dipper time in type 2 diabetic patients. Thiazolidinediones, such as rosiglitazone and PG, belong to a class of insulin-sensitizing medications that mediate their metabolic effects through the ligand-activated nuclear transcription factor PPAR-*γ*. Thiazolidinediones improve metabolic derangements and exert beneficial effects in diabetic patients [17, 18]. The PPAR-*γ* agonist rosiglitazone reversed the elevated expression of clock genes mRNA in insulin-resistant mice [19]. Such data strongly indicate the essential role of PPAR-*γ* in maintaining circadian rhythms of blood pressure and heart rate, which may partially explain the beneficial side effects of PPAR-*γ* agonists in the cardiovascular system. These findings demonstrated a novel role of PPAR-*γ* as a regulator in correlating circadian rhythm and metabolism [20].

This study examined the influence of PPAR-*γ* agonist PG on the mRNA expression profile of principle clock genes and inflammation-related genes in the mouse liver disrupted by reversed feeding.

## 2. Materials and Methods

### 2.1. Animals

**Figure 1:**
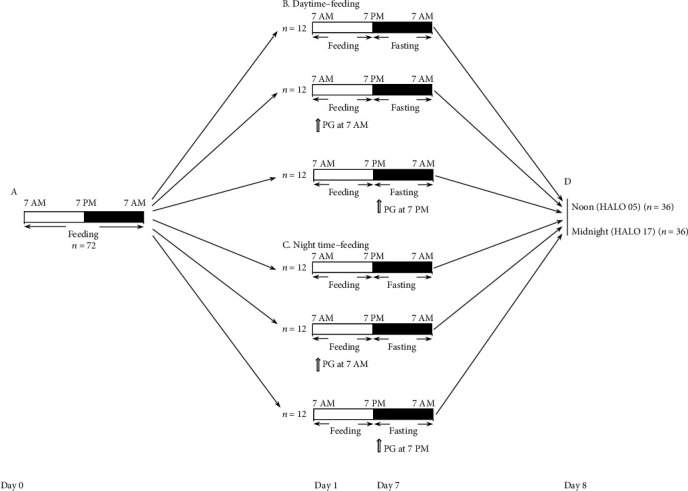
Experimental flowchart. Open and solid bars indicate light and dark periods, respectively. Solid lines indicate feeding and fasting periods. Experimental schedule from day 0 to day 8: (a) mice entrained to a 12-h light-dark cycle with ad libitum access to food and water; (b) mice in daytime-feeding groups; and (c) mice in nighttime-feeding groups. (d) Sacrification was performed at day 8 noon (HALO 05) and midnight (HALO 17), and liver specimens and serum samples were collected. Sacrification for histopathological analysis and Western blotting was performed at HALO 05. White arrows indicate the time of pioglitazone administration.

Eight-week-old BALB/c male mice were housed individually in single cages to avoid aggression on a 12 : 12 light-dark cycle (lights on at 7 AM, lights off at 7 PM), with food and water available ad libitum. The study was approved by the Ethics Committee of Poltava State Medical University. Mice were randomly assigned to daytime-feeding and nighttime-feeding groups. Mice in daytime-feeding groups received food from 7 AM to 7 PM, and mice in nighttime-feeding groups received food from 7 PM to 7 AM [21]. PG was administered in the dose of 20 mg/kg per os as aqueous suspension 40 *μ*l at 7 AM or 7 PM. Each group consisted of 12 animals. All manipulations at the dark phase were provided under red light.  Figure 1 shows the study design.

On day 8 of the feeding intervention, mice were sacrificed by cervical dislocation at noon (05 hours after light onset (HALO)) and midnight (HALO 17). The livers were removed, and part of the left lateral lobe samples was immediately frozen and kept at -80°C until use. Blood samples were collected from the right ventricles, and sera were obtained by centrifugation.

### 2.2. RNA Preparation and Quantitative Reverse Transcription PCR

Six mice at each of two timepoints (HALO 05 and HALO 17) were used for RNA extraction. Left lateral liver lobe samples were collected. Total RNA was extracted from the liver using RNA easy kit (QIAGEN, USA).

To generate single-strand DNAs, total RNA (≈1 *μ*g) was reverse transcribed using QuantiTec®Reverse Transcription Kit (QIAGEN, USA).

For SYBR Green-based analysis, the cDNA equivalent of 50 ng of total RNA from each sample was amplified in the CFX96TM Real-Time PCR Detection System (BIO-RAD, USA) using a QuantiTec®SYBR-Green I PCR Kit (QIAGEN, USA).

Each sample was analyzed in duplication to ensure the accuracy of the data. The gene expressions were detectable as 2^–*Δ*CT^. All the values were normalized to the expression of the housekeeping gene *β*-actin.

The sequences of specific primers used for real-time PCR are provided in Table 1.

### 2.3. Western Blot Analysis

Liver tissues were homogenized in lysis buffer containing phenylmethylsulfonyl fluoride in TissueLyser LT (QIAGEN, USA) homogenizer. The homogenate was centrifuged at 12.000 rpm for 20 min at 4°C. After total protein level adjustment, the samples were denaturated in loading buffer for 10 min at 95°C. Samples (40 *μ*g of protein) were separated in 7.5% SDS-PAA gel and then transferred onto nitrocellulose membranes Immobilon® HAHY (Sigma-Aldrich, USA). Membranes are blocked with 5% fat-free dried milk for 2 hours at room temperature and incubated overnight at 4°C with rabbit anti-PPAR-*γ* antibody (SAB4502262, 1 : 1000, Sigma-Aldrich, USA), rabbit anti-pNF-*κ*B p65 antibody (SAB4301496, 1 : 1000, Sigma-Aldrich, USA), rabbit anti-IL-6 antibody (SAB5700632, 1 : 1000, Sigma-Aldrich, USA), and rabbit anti-*β*-actin antibody (ZRB1312, Sigma-Aldrich, USA). After the washing, membranes were incubated with antirabbit IgG-Biotin (B8895), streptavidin, and HRP conjugate (18.152). The immunoreactive bands were stained using AEC Staining Kit (Sigma-Aldrich, USA).

### 2.4. Serum Measurement

Glucose, total cholesterol, triglyceride concentrations, alanine aminotransferase (ALT), and asparagine aminotransferase (AST) activities were measured in sera by the photometric method using kits Erba® Mannheim. The oxidase activity of ceruloplasmin was determined spectrophotometrically according to the method described by Sunderman and Nomoto [24], with p-phenylenediamine as a substrate.

### 2.5. Histological Analysis

For histological analysis, fixed liver samples (left lateral liver lobes) were embedded in paraffin blocks. Sections (4-5 *μ*m) were cut on a microtome and stained with hematoxylin and eosin. Morphological and morphometrical analyses were performed using light microscopy. The proportion of various hepatic tissue components was determined by microscope Axio Lab.A1 (Carl Zeiss, Göttingen, Germany) software. Software program ZEN 2.5 (blue edition) version V1.0 04/2018 103 was used for cell counting and measurement of the mean diameter of hepatocyte nuclei.

The minimal set of staining included hematoxylin and eosin (H&E) and Malory's staining. All biopsies were reviewed by the same pathologist. For each biopsy, a pre-established form for evaluation of the main histologic patterns was filled out by the pathologist blinded to experimental group affiliation of biopsies.

The histological checklist included is as follows: (1) assessment of the organization of hepatocellular cords and sinusoids (0, normal; or 1, damaged); (2) measurement of the mean diameter of hepatocyte nuclei in zone 2 (for the 5 largest nuclei); (3) counting of nonparenchymal elements (NPE) (lymphocytes, Kupffer cells, and Ito cells) [25] on the area of 1000 *μ*m^2^ in 5 fields of view ×400 (HPF); and (4) assessment of liver cell ballooning from 0 to 3 (0, normal hepatocytes with cuboidal shape and pink eosinophilic cytoplasm; 1, the presence of clusters of hepatocytes with a rounded shape and pale cytoplasm usually reticulated). Although the shape is different, the size is quite similar to that of normal hepatocytes; 2 is the same as grade 1 with some enlarged hepatocytes, at least 2-fold that of normal cells; and 3 is similar to 2, but almost all hepatocytes changed. (5) Lobular inflammation was defined as a focus of two or more inflammatory cells within the lobule. Foci were counted at 200× magnification (0, none; 1, ≥2 foci per 200× field); (6) portal inflammation was assessed from low magnification (0, none to min; 1, greater than min); (7) pigmented macrophages were assessed from 400× magnification similar to previous, 0/1; and (8) changes in the microcirculation were described as a sludge of erythrocytes (0, none to min; 1, greater than min); stasis of erythrocytes (0, none to min; 1, greater than min); and the phenomena of plasmostasis or cholestasis (0, none to min; 1, greater than min).

### 2.6. Statistical Analysis

Statistical analysis was performed using GraphPad Prism 5.0 (GraphPad Software, San Diego, USA) using descriptive statistics, one-way ANOVA, and post-hoc Bonferroni test with a further calculation of Mann–Whitney test for unpaired samples. 2 × 2 table statistic, including Fisher's exact test, was used to compare proportions. *P* values of <0.05 were considered statistically significant in all of the analyses.

The null hypothesis tested was that PG and the time of its administration have no influence on the clock and inflammation-related gene expression is impaired by reversed feeding.

## 3. Results

### 3.1. Effects of Feeding Intervention and PG Intake on Clock Gene Expression in Mouse Liver

**Figure 2:**
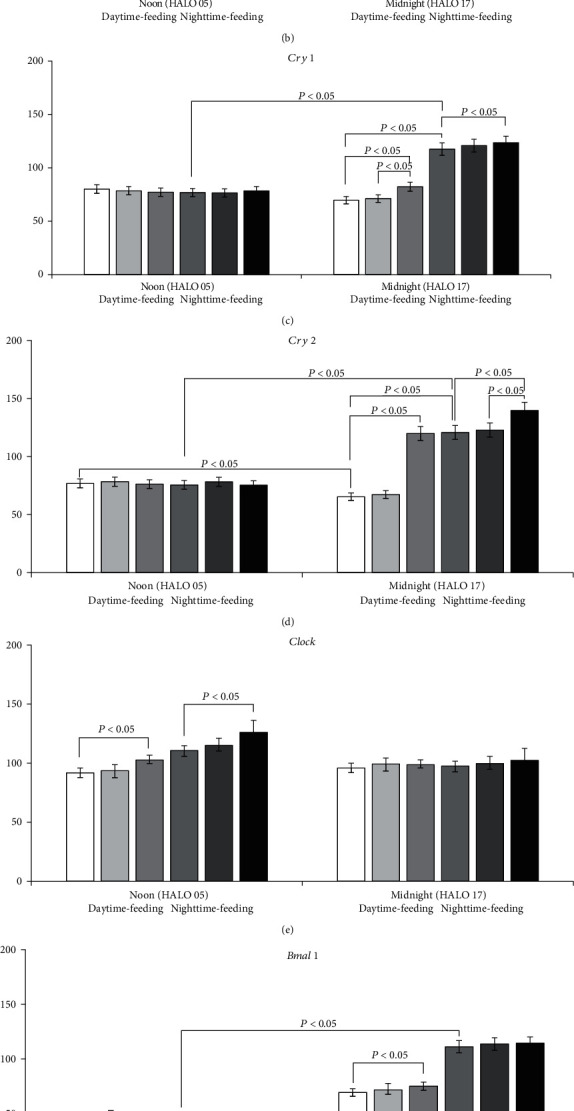
Circadian changes in the mouse clock gene mRNA transcription in the liver tissues. Expression of mRNA: (a) *Per 1*; (b) *Per 2*; (c) *Cry1*; (d) *Cry2*; (e) *Clock*; (f) *Bmal1*; and (g) *Rev-erb alpha*. Significant differences are shown by horizontal lines (*P* < 0.05).

Figure 2 shows the expression pattern of clock genes in mouse liver on the 8th day after initiation of the feeding intervention.

In the nighttime-feeding group, expression of *Per1*, *Per2*, *Cry1*, *Cry2*, and *Bmal1* was statistically significantly higher at midnight (HALO 17) than at noon (HALO 05) (*P* < 0.05). At the same time, the expression of *Rev-erb alpha* was higher at noon (HALO 05). Administration of PG at 7 AM did not influence the clock gene expression. In contrast, PG intake at 7 PM slightly increased the *Cry1* and *Cry2* expression at midnight and the *Clock* expression at noon (*P* < 0.05).

The daytime feeding led to important changes in gene expression. Expression of *Per1* and *Per2* had another pattern with a decreased level at midnight and an increased one at noon (*P* < 0.05). In addition, we observed a decrease in *Cry1* and *Cry2* expression at midnight in comparison with nighttime feeding. A similar reverse pattern was observed for *Rev-erb alpha* expression with an increased level at midnight and a decrease at noon (*P* < 0.05).

PG intake at 7 AM led to an increase in *Per2* expression at midnight and *Rev-erb alpha* at noon. Administration of PG at 7 PM induced an increase in *Per1*, *Per2*, *Cry1*, *Cry2*, and *Bmal1* expressions at midnight and a decrease in *Per1* and *Per2* expression at noon. *Rev-erb alpha* expression decreased at midnight and demonstrated a further increase at noon (*P* < 0.05).

### 3.2. Effects of Feeding Intervention and PG Intake on Inflammation-Related Gene Expression in Mouse Liver

**Figure 3:**
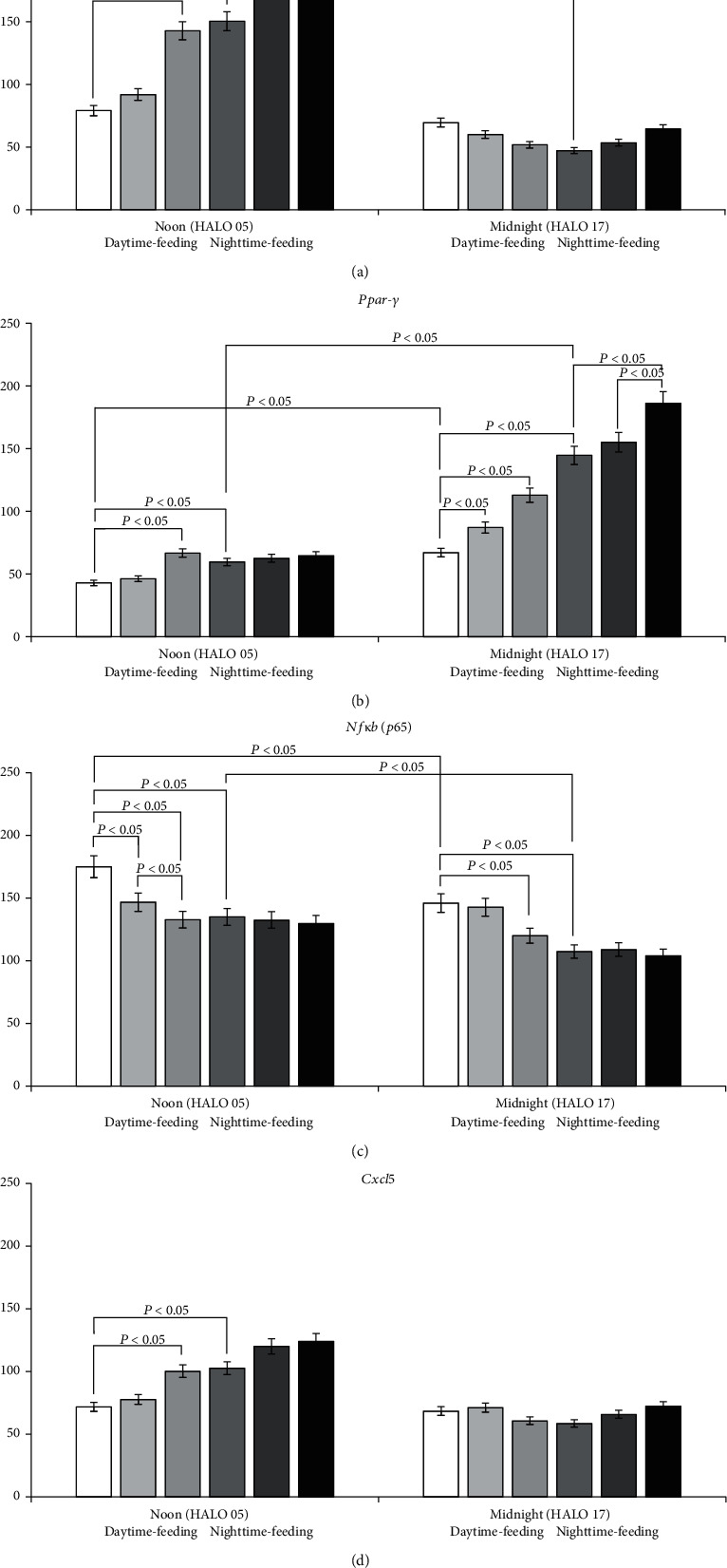
Circadian changes of mRNA transcription of inflammation-related genes in mouse liver tissues. mRNA expression of (a) *Nrf2*; (b) *Ppar-γ*; (c) *Nfκb* (*p65*); and (d) *Cxcl5*. Significant differences are shown by horizontal lines (*P* < 0.05).

Figure 3 shows the expression pattern of inflammation-related genes in mouse liver after feeding intervention.

In the nighttime-feeding group, the expression of *Nrf2* and *Nfκb* was significantly lower at midnight than at noon, and expression of *Ppar-γ* was higher at midnight (*P* < 0.05).

The daytime feeding induced statistically significant differences in gene expression. There was no difference between the midnight and noon expression of *Nrf2*. We observed a significant decrease in *Nrf2* expression at noon in comparison with the nighttime-feeding group.

In addition, a significant decrease in *Ppar-γ* expression was observed at midnight and noon in comparison with nighttime-feeding mice. In contrast, a significant increase in *Nfκb* expression was observed at midnight and noon in comparison with the nighttime-feeding group. There were no differences in *Cxcl5* expression.

PG administration at 7 AM to nighttime-feeding mice did not influence gene expression significantly. We received important data that PG intake at 7 PM increased *Nrf2* and *Ppar-γ* expression at noon. Administration of PG at 7 AM to daytime-feeding group led to an increase in *Ppar-γ* expression at midnight and a decrease in *Nfκb* expression at noon. PG intake at 7 PM led to an increase in *Ppar-γ* expression at midnight and a decrease at noon (*P* < 0.05). At the same time, we observed an increase in *Nrf2* and *Cxcl5* expression and a decrease in *Nfκb* expression at noon.

### 3.3. Effect of Feeding Intervention and PG Intake on Biochemical Substrates in Mouse Sera

Table 2 summarizes the effects of feeding intervention and PG intake on biochemical substrates in mouse sera.

In the nighttime-feeding group, no statistically significant differences were observed in the levels of glucose, ceruloplasmin, triglycerides, total cholesterol, and in the activity of ALT and AST. The daytime feeding induced an increase in the levels of glucose, ceruloplasmin, triglycerides, total cholesterol, and the activity of ALT and AST (*P* < 0.05). Administration of PG at 7 AM or at 7 PM to nighttime-feeding animals did not reveal any influences on substrates investigated. In the daytime-feeding group, PG intake at 7 AM decreased only the level of AST activity. In contrast, we observed decreased glucose, ceruloplasmin, total cholesterol levels, and activities of ALT and AST after PG intake at 7 PM (*P* < 0.05).

The aforementioned results demonstrated that reversed feeding induced the changes in the expression of clock and inflammation-related genes, and PPAR-*γ* agonist PG also influenced this expression. Therefore, we investigated the effect of reversed feeding and the PPAR-*γ*/NF-*κ*B/IL-6 inflammatory axis in mouse liver.

**Figure 4:**
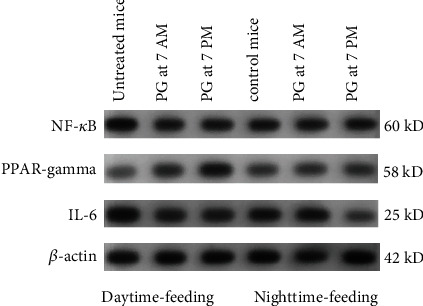
Western blot results of PPAR-*γ*, pNF-*κ*B, and IL-6 expression in the liver tissue of experimental animals.

As depicted in Figure 4, Western blotting results show that compared with the control group (nighttime feeding), the expression of PPAR-*γ* in the liver of the daytime-feeding group decreased significantly. In contrast, daytime feeding led to a remarkable increase in pNF-*κ*B and IL-6 expression in liver tissue. PG administration at 7 AM or at 7 PM to nighttime-feeding mice did not influence the expression of PPAR-*γ*, pNF-*κ*B, or IL-6. When PG was administered to daytime-feeding mice at 7 AM, we observed a slight decrease in pNF-*κ*B and IL-6 expression. Administration of PG at 7 PM decreases the expression of pNF-*κ*B and IL-6 to the level of control mice.

### 3.4. Effect of Feeding Intervention and PG Intake on Mouse Liver Histology

The histopathological analysis did not show degenerated areas, fibrosis, steatosis, or microsteatosis in experimental animals. Ballooning and microcirculation demonstrated noticeable, but not statistically significant alterations when compared to the control group.  Table 3 summarizes the effects of feeding intervention and PG intake on mouse liver histology.

**Figure 5:**
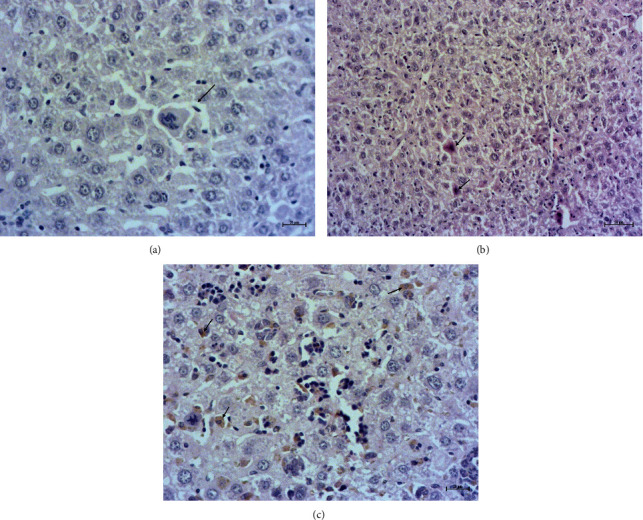
Intrahepatocellular edema (a) and acidophil bodies (b) (arrows) in the samples of *daytime-feeding untreated* animals. Pigmented macrophages (c) in the sample of liver of *daytime-feeding PG* at 7 AM animals. H&E staining; mg. ×400, ×200, and ×400.

In the *daytime-feeding untreated* animals, a prominent finding was intrahepatocellular edema at 7 out of 13 preparations (Figure 5(a)). In addition, the acidophil bodies (Figure 5(b)) were significantly more often presented, as compared to the control (*P* < 0.05) and *daytime-feeding PG* at 7 PM groups (*P* < 0.05), as shown by Fisher's exact test. Therefore, PG intake at 7 PM in the *daytime-feeding animals* decreased significantly the frequency of the acidophil bodies.

**Figure 6:**
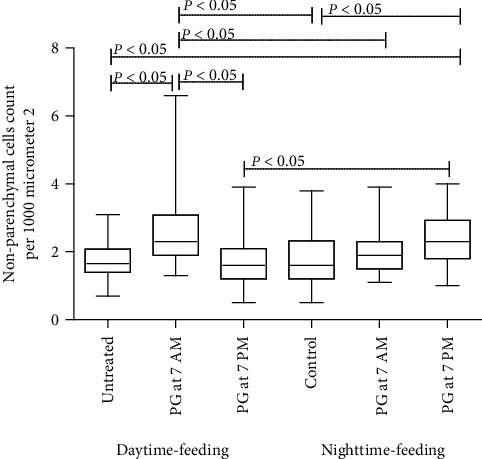
Comparison of nonparenchymal cell counts in the liver tissue of experimental animals.

The levels of NPE infiltration did not differ significantly between daytime- or nighttime-feeding untreated groups. However, PG intake led to a rise in NPE inside both the daytime-feeding and the nighttime-feeding groups. PG intake at 7 AM in the daytime-feeding animals led to one of the highest levels of NPE infiltration (Figure 6). Occasionally, one of these animals demonstrates pigmented macrophages as shown in Figure 5(c).

The hepatic parenchyma arrangement is presented by “acinus” or “lobular” regions, which are interspersed with central venules and portal tracts. Portal tracts contain a portal vein, a branch of the hepatic artery, and a bile duct. Three different “zones” are defined by these structures: Zone 1 is adjacent to the portal tract, zone 3 is closest to the hepatic venule, and zone 2 is in between [26]. We measured the nuclei in zone 2, where they usually are the largest.

**Figure 7:**
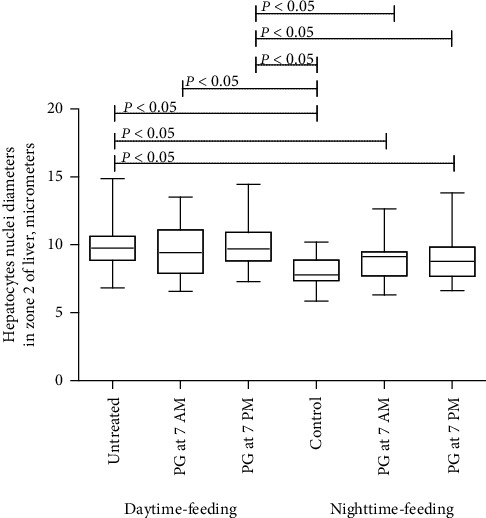
Comparison of the nuclear diameter of hepatocytes between the groups.

In daytime-feeding untreated animals, a significant increase in the nuclear diameter of hepatocytes was observed. PG intake at 7 PM led to the same effect. But PG intake at 7 AM differed significantly only from the control nighttime-feeding untreated group (Figure 7). Despite the absence of significant differences within the daytime or nighttime-feeding groups, the revealed trend seemed to balance all the groups taking PG.

According to other histological checklist points, no statistically significant differences were found between the groups.

## 4. Discussion

Human homeostatic systems have adapted to daily changes in a way that the body anticipates the sleep and activity periods. The endogenous circadian clock is located in the suprachiasmatic nucleus of the anterior hypothalamus that responds to solar time. Similar clocks have been found in peripheral tissues, such as the liver [27, 28]. Shifting the light-dark cycle alters the rhythms of master clock genes, and changing the feeding rhythm alters the rhythms of peripheral clock genes. This alteration led to the increased responses to lipopolysaccharide, suppression of natural killer cell cytotoxic activity, impaired reactive oxygen species production and its elimination, and detoxication disorders [29–31]. The uncoupling of peripheral and master clock gene rhythms by reversed feeding led to an exacerbated inflammatory response after polymicrobial sepsis or lethal effect of lipopolysaccharide [7, 32]. Important data were received that upon nutrient challenge the liver-clock attenuates the effect of feeding-related signals on rhythmicity of other peripheral tissues, irrespective of their own clocks [33].

The reversed feeding might impair the temporal expression patterns of proteins that form interlocking transcription-translation feedback loops consisting of seven recognized key genes— *Per1*, *Per2*, *Cry1*, *Cry2*, *Rev-erb alpha*, *Clock*, and *Bmal1*.

Our results demonstrated that mice at daytime feeding had significantly lower liver *Per1*, *Per2*, and *Cry2* expression at midnight (HALO 17) than at noon (HALO 05). In contrast, the expression of *Rev-erb alpha* was increased at noon. These data went in parallel with other observations [22, 34]. The reversed feeding influenced the liver clock gene expression dramatically: Expression of *Per1*, *Per2*, *Cry1*, and *Rev-erb alpha* increased at noon. There were no differences in the temporal expression of *Clock* and *Bmal1*. Similar results that daytime feeding induced clock genes uncoupling were received in a previous study [7, 35, 36].

Simultaneously, we investigated expressions of several inflammation-related genes— *Nrf2*, *Ppar-γ*, *Nfκb* (*p65*), and *Cxcl5*. Nighttime-feeding mice had an increased expression of *Nrf2* and *Nfκb* (*p65*) at noon in comparison with the expression at midnight. *Ppar-γ* had another expression profile with an increased expression at midnight. These findings correlated with previously described data for liver tissue [19, 37, 38]. The reversed feeding canceled the circadian pattern of *Nrf2* expression, decreased the midnight expression of *Ppar-γ*, and increased the midnight expression of *Nfκb* (*p65*). The expression of *Cxcl5* did not vary significantly. Thus, reversed feeding influenced the changes in the peripheral clock gene rhythm in the mouse liver tissue accompanied by a decreased expression of *Nrf2* and *Ppar-γ* and an increased expression of *Nfκb* (*p65*).

These transcriptional factors have a complicated interaction network between each other and with molecular components of the circadian clock. Nrf2 regulates the core and stabilizes circadian clock loops, coupling redox, and timekeeping. Mouse liver cells need Nrf2 to maintain a normal circadian rhythm, and a closer inspection of the liver cells revealed that Nrf2 specially attaches to part of the gene for a clock protein Cry2. In this way, Nrf2 links metabolism signals to the ticking of the circadian clock [39].

Clock/Bmal1-dependent Nrf2 regulation gives rise to diurnal patterns in Nrf2 signaling, which underlies the rhythmic expression of antioxidant and metabolic enzymes. In the mouse knockout model, Bmal/absence in hepatocytes caused the activation of redox sensor Nrf2 [34, 40].

PPAR-*γ* is a regulator of inflammation and circadian rhythm [41]. On the other hand, Per2 might control the activity of PPAR-*γ* by operating as its natural modulator. Per2 exerts its repressive action on PPAR-*γ* by blocking its recruiting to target promoters, a mechanism that is conceptually different from the repression that Per2: Cry exerts on Clock:Bmal1 [42].

*Rev-erb alpha*, a target gene of PPAR-*γ*, is one of the core clock components, although there has not been direct evidence showing that PPAR-*γ* exerts circadian function via Rev-erb alpha [43].

NF-*κ*B responses to a variety of immunomodulators are mediated by core circadian protein Clock, which can upregulate NF-*κ*B-mediated transcription in the absence of Bmal1. Moreover, Bmal1 counteracts the Clock-dependent increase in the activation of NF-*κ*B-responsive genes. The clock is found in a protein complex with the p65 subunit of NF-*κ*B, and its overexpression correlates with an increase in specific phosphorylated and acetylated transcriptionally active forms of p65 [44].

In daytime-feeding mice, there were increased levels of glucose, ceruloplasmin, lipids-triglycerides, and total cholesterol. In these mice, there was liver cytolysis with a preferential increase in ALT activity. These data supported that clock disruption led to impaired glucose metabolism and insulin signaling [45] as well as to lipid disorders [46]. Ceruloplasmin elevation might be mediated through the activation of NF-*κ*B pathway [47]. Our investigation showed that daytime feeding led to an increase in pNF-*κ*B and IL-6 levels, displaying the proinflammatory shift in the PPAR-*γ*/NF-*κ*B/IL-6 axis. These data went in parallel with the observation that hepatocytes, in addition to immune cells, might also serve as a direct source of IL-6 and that IL-6 mRNA production occurred under the transcriptional control of NF-*κ*B [48, 49]. Moreover, Norris CA et al. showed that the synthesis of IL-6 by hepatocytes was a normal response to common hepatic stimuli [50].

In daytime-feeding mice, we observed intercellular edema and an increased level of acidophilic bodies in the liver tissues, along with a significant increase in the nuclear diameter of hepatocytes. These data went in parallel with the observation that the circadian clock plays critical roles in mediating several hepatic functions under physiological conditions, and whose deregulation is implicated in chronic liver diseases including nonalcoholic steatohepatitis and alcohol-related liver disease [51]. The enlargement of hepatocyte nuclei might display an intensive protein-DNA interaction [52]. There are clear effects of nuclear shape on the transcriptional activity of the cell. A recent study has shown that the regulation of gene expression can be influenced by nuclear morphology and that cells can drastically remodel their chromatin during differentiation [53]. The protein component of the nuclear envelope is the focus of many studies, which demonstrated that chromatin–proteins interaction affected the stiffness of the nucleus [54], and determined the shape of the nucleus internally [55]. However, the lipid component is also relevant. Defects in the lipids can lead to overproduction of membrane lipids and expansion of the nuclear membrane [56], which were demonstrated as increased nuclear size, involutions, and projections from the nuclear envelope observed in both yeasts [57] and *Drosophila melanogaster* [58].

As the next part of our study, we examined the influence of PPAR-*γ* agonist PG on the mRNA expression profile of principle clock genes and inflammation-related genes disrupted by reversed feeding in the mouse liver.

Recently, the ability of PPAR-*γ* agonists to influence clock gene expression was suggested. Ribas-Latre A. et al. (2019) showed that rosiglitazone restored changes in high fat diet-induced Bmal1 recruitment and activity in mouse liver [59]. Nevertheless, there is a lack of information about chronopharmacological aspects of PPAR-*γ* agonist activity.

Administration of PG at 7 AM to nighttime-feeding mice did not reveal any influence on the expression of the clock or inflammation-related genes either at midnight or at noon. In the daytime-feeding group, PG intake at 7 PM led to an increase in *Per2* and *Rev-erb alpha* mRNA at noon, an increase in *Ppar-γ* mRNA at midnight, and a decrease in *Nfκb* (*p65*) mRNA at noon. In general, PG administration at 7 PM slightly normalized the impaired expression of clock genes and increased anti-inflammatory potency impaired by reversed feeding. This pattern was supported by biochemical substrate levels such as glucose and total cholesterol. The decrease in NF-*κ*B led to the inhibition of ceruloplasmin levels. In the daytime-feeding group, we observed that PG intake at 7 PM led to an increase in PPAR-*γ* level and a decrease in pNF-*κ*B and IL-6 levels in liver tissues. Our data support the previous report [60] that rosiglitazone activated PPAR-*γ* to inhibit the NF-*κ*B inflammatory axis.

According to our data, PG intake at 7 PM exerts strong normalization of clock gene expression with the further increase in *Nrf2* and, especially, *Ppar-γ* expression with inhibition of *Nfκb* expression in the liver of daytime-feeding mice. These expression changes went in parallel with the decreasing hyperglycemia, hypercholesterolemia, and ALT and AST activities. One important note might be a clone that PG has extrahepatic effects and might influence metabolism in this model. Thus, PG has a chronopharmacological effect and might be administered at 7 PM.

The first data about the ability of PG to influence the circadian rhythm of blood pressure were received in type 2 diabetic patients [17]. The normalizing effect on the clock observed in mice (which may also be occurring in patients taking thiazolidinediones) may be due to some interplay between Bmal1 and PPAR-*γ* proteins at the level of chromatin function [61, 62]. PPAR-*γ* ligands attenuate NF-*κ*B signaling [63]. For example, rosiglitazone suppresses LPS-mediated inflammation via Nrf2, and PPAR-*γ* activation [64] attenuates NF-*κ*B/p65 activation and Nox4 expression during high glucose-induced oxidative stress [65]. Also, PPAR-*γ* is a transcriptional activator of mouse cytochrome P450 2a5, and its rhythmic expression contributed to the circadian efficacy of the detoxication system in the liver [66].

There is evidence of a strong connection between meal timing, the liver clock, metabolic homeostasis, and nonalcoholic fatty liver disease (NAFLD) with further liver inflammation, fibrosis, cirrhosis, and hepatocellular carcinoma [67]. Sleep disruption, daytime sleepiness, sleep quality, and inactivity correlate with the severity of NAFLD in patients [68–70].

Little is known about the influence of the feeding time and reversed feeding on liver functions and morphology. Recent data showed that there were cellular morphological rhythms in the fish liver as well as the interactions between light and feeding cycles in the different metabolic zones of the liver [71].

It is important to emphasize that PPAR-*γ* is currently under study as a drug target in NAFLD and is modulated by circadian proteins [72, 73]. The circadian clock itself has the potential for use as a target for the treatment of NAFLD [74, 75]. Thus, it was important to investigate the histopathological differences in mice livers induced by reversed feeding as well as by daytime and nighttime PG dosing.

According to our histology data, PG intake also had an impact on the mouse liver, manifested as an increase in the number of nonparenchymal cells, but the contribution of these cells to the circadian rhythmicity of the liver functions is still unclear [76]. In addition, despite the statistically insignificant data about restoring the increased hepatocytes nuclear size, PG at 7 PM was favorable for reversed feeding mice. This finding probably reflects that PG enhanced protein-chromatin interaction especially when administered in the evening.

Taking together, our data support the assumption that reversed feeding disrupted mouse liver circadian expression pattern of clock genes and genes involved in the metabolism and inflammation regulation (*Nrf2*, *Ppar-γ*, and *Nfκb*) with impaired glucose and lipid metabolism, increased activities of ALT and AST, and liver histopathological changes. Administration of PPAR-*γ* agonist PG restored the circadian expression pattern, especially when PG was admitted at 7 PM. This restoration was in parallel with the normalization of glucose and lipid levels in the blood and liver morphology.

The limitation of this study might be the extrahepatic effects of PG and their influence on metabolism in this model as well as we cannot discriminate the levels of pNF-*κ*B/IL-6 in hepatocytes or in Kupffer cells. The duration of the experimental model might be insufficient for the development of more significant changes in experimental animals. The obtained results cannot be transferred to clinical practice. Prospects for future research might be further investigation of inflammatory cytokines and insulin resistance in reversed feeding mice and in other animal models with the uncoupling of peripheral and master clock gene rhythm. Additional investigation might be provided to exclude extrahepatic effects of PG and their influence on metabolism in this model. Further pilot clinical observations are needed to estimate the chronopharmacological properties of PG in type 2 diabetes patients.

## 5. Conclusions

Our study indicates that reversed feeding induced the disruption of mouse liver circadian expression pattern of clock genes accompanied by increasing *Nfκb* and pNF-*κ*B, IL-6 expression, and decreasing *Nrf2* and PPAR-*γ*.

Administration of PG restored the clock gene expression profile and decreased *Nfκb*, pNF-*κ*B, and IL-6, as well as increased *Nrf2*, *Ppar-γ*, and PPAR-*γ* expression. PG intake at 7 PM was more effective than at 7 AM.

## Figures and Tables

**Table 1:** Primer sequences for mRNA measurement.

Gene	Primer sequences	References
*Bmal1*	Forward ACATAGGACACCTCGCAGAA	[22]
	Reverse AACCATCGACTTCGTAGCGT	
*Clock*	Forward CCTATCCTACCTTCGCCACACA	
	Reverse TCCCGTGGAGCAACCTAGAT	
*Rev-erb alpha*	Forward CGTTCGCATCAATCGCAACC	
	Reverse GATGTGGAGTAGGTGAGGTC	
*Cry1*	Forward TTGCCTGTTTCCTGACTCGT	
	Reverse GACAGCCACATCCAACTTCC	
*Cry2*	Forward TCGGCTCAACATTGAACGAA	
	Reverse GGGCCACTGGATAGTGCTCT	
*Per1*	Forward CATGACTGCACTTCGGGAGC	
	Reverse CTTGACACAGGCCAGAGCGTA	
*Per2*	Forward GGCTTCACCATGCCTGTTGT	
	Reverse GGAGTTATTTCGGAGGCAAGTGT	
*Cxcl5*	Forward TGCCCTACGGTGGAAGTCAT	[13]
	Reverse AGCTTTCTTTTTGTCACTGCCC	
*Nrf2*	Forward AAGCTTTCAACCCGAAGCAC	[23]
	Reverse TTTCCGAGTCACTGAACCCA	
*Ppar-γ*	Forward CCAGAGCATGGTGCCTTCGCT	
	Reverse CAGCAACCATTGGGTCAGCTC	
*Nfκb (p65)*	Forward GAGGTCTCTGGGGGTACCAT	
	Reverse AAGGCTGCCTGGATCACTTC	
*β*-Actin	Forward ACTGCCGCATCCTCTTCCTC	
	Reverse CTCCTGCTTGCTGATCCACATC	

**Table 2:** Effects of feeding intervention and PG intake on the level of biochemical substrates in mouse sera.

Indices	Time of feeding					
	Daytime			Nighttime		
	Untreated mice (*n* = 12)	PG at 7 AM (*n* = 12)	PG at 7 PM (*n* = 12)	Control mice (*n* = 12)	PG at 7 AM (*n* = 12)	PG at 7 PM (n =12)
Glucose, mmol/l	10.27 (5.70-11.34)	8.99 (6.06-9.36)	7.42 (2.27-7.73)	3.09 (2.98-8.85)	3.28 (2.96-7.46)	2.47 (2.25-6.12)
	*P*_1_ = 0.0147	*P*_2_ = 0.0355	*P*_4_ = 0.0433			
	*P*_2_ = 0.0147					
	*P*_3_ = 0.0052	*P*_3_ = 0.0185				
Ceruloplasmin, mg/l	243.04 (140.88-271.50)	229.51 (105.00-268.63)	148.44 (91.38-164.63)	129.25 (113.75-148.75)	134.94 (101.50-154.88)	122.50 (107.63-155.75)
	*P*_1_ = 0.0342		*P*_4_ = 0.0232			
	*P*_2_ = 0.0185					
	*P*_3_ = 0.0255					
Triglycerides, mmol/l	1.42 (1.03-1.48)	0.84 (0.77-1.31)	0.79 (0.77-1.05)	0.85 (0.52-1.16)	0.83 (0.66-1.06)	0.76 (0.57-0.85)
	*P*_1_ = 0.0140					
	*P*_3_ = 0.0112					
Total cholesterol, mmol/l	3.52 (2.09-3.88)	2.90 (1.68-3.51)	2.07 (1.79-2.91)	1.70 (1.61-2.96)	1.85 (1.53-3.18)	1.56 (1.46-2.87)
	*P*_1_ = 0.0052		*P*_4_ = 0.0232			
	*P*_2_ = 0.0493					
	*P*_3_ = 0.0029					
ALT, U/l	113.40 (99.15-121.45)	107.10 (95.65-116.35)	81.45 (74.54-91.60)	64.60 (62.67-66.85)	69.87 (64.66-73.30)	72.91 (69.85-75.54)
	*P*_1_ < 0.05	*P*_1_ < 0.05	*P*_4_ < 0.05			
	*P*_2_ < 0.05	*P*_2_ < 0.05	*P*_5_ < 0.05			
	*P*_3_ < 0.05	*P*_3_ < 0.05				
AST, U/l	107.51 (95.84-113.18)	98.05 (88.79-102.16)	87.00 (78.14-92.01)	79.78 (75.84-84.31)	75.91 (70.95-86.91)	89.04 (83.88-93.75)
	*P*_1_ < 0.05	*P*_1_ < 0.05	*P*_4_ < 0.05			
	*P*_2_ < 0.05	*P*_2_ < 0.05	*P*_5_ < 0.05	*P*_3_ < 0.05	*P*_3_ < 0.05	
	*P*_3_ < 0.05	*P*_4_ < 0.05				

*P* value: *P*_1_: when compared with control mice (nighttime); *P*_2_: when compared with PG at 7 AM (nighttime); *P*_3_: when compared with PG at 7 PM (nighttime); *P*_4_: when compared with untreated mice; and *P*_5_: when compared with PG at 7 AM (daytime).

**Table 3:** Histopathological and morphometrical data of liver tissue of experimental animals.

Histological checklist	Time of feeding					
	Daytime			Nighttime		
	Untreated mice (*n* = 12)	PG at 7 AM (*n* = 8)	PG at 7 PM (*n* = 10)	Control mice (*n* = 10)	PG at 7 AM (*n* = 9)	PG at 7 PM (*n* = 10)
Hepatocellular cords and/or sinusoid destruction, cases per group	0 (0.0%)	0 (0.0%)	1 (1.0%)	1 (1.0%)	1 (11.1%)	0 (0.0%)
Diameter of hepatocyte nuclei in zone 2, *μ*m, *М* ± SD	9.82 ± 1.58	9.59 ± 1.90∗	10.04 ± 1.77∗	7.99 ± 1.04∗	8.88 ± 1.24	8.86 ± 1.42
Nonparenchymal cells count per 1000 *μ*m^2^, *М* ± SD	1.73 ± 0.56∗	1.23 ± 0.19∗	1.69 ± 0.73∗	1.74 ± 0.78∗	2.01 ± 0.68	2.32 ± 0.72∗
Liver cell injury: ballooning, cases per group	8 (66.7%)	6 (75.0%)	10 (100.0%)	6 (60.0%)	8 (88.9%)	7 (70.0%)
Liver cell injury: acidophil bodies, cases per group	7 (58.3%)∗∗	4 (50.0%)	2 (20.0%)∗∗	0 (0.0%)∗∗	2 (22.2%)	0 (0.0%)
Liver cell injury: pigmented macrophages, cases per group	0 (0.0%)	1 (12.5%)	0 (0.0%)	0 (0.0%)	0 (0.0%)	0 (0.0%)
Lobular inflammation, cases per group	5 (41.7%)	5 (62.5%)	3 (30.0%)	4 (40.0%)	4 (44.4%)	8 (80.0%)
Portal inflammation, cases per group	0 (0.0%)	1 (12.5%)	0 (0.0%)	1 (10.0%)	0 (0.0%)	1 (10.0%)
Circulation alteration: cases of sludge per group	8 (66.7%)	4 (50.0%)	6 (60.0%)	3 (30.0%)	2 (22.2%)	4 (40.0%)
Circulation alteration: cases of stasis per group	11 (91.7%)	8 (100.0%)	10 (100.0%)	9 (90.0%)	8 (88.9%)	9 (90.0%)
Circulation alteration: cases of plasmostasis per group	7 (58.3%)	5 (62.5%)	7 (70.0%)	4 (40.0%)	4 (44.4%)	5 (50.0%)

*P* value calculated by one-way ANOVA and post-hoc Bonferroni tests ∗ and Fisher's exact test ∗∗ statistically significant differences (*P* < 0.05).

## Data Availability

All the data on clock gene expression and inflammation-related genes used to support the findings of this study are included within the article.
